# Redistributing ill-defined causes of death – a case study from the BURDEN 2020-project in Germany

**DOI:** 10.1186/s13690-021-00535-1

**Published:** 2021-03-15

**Authors:** Annelene Wengler, Heike Gruhl, Dietrich Plaß, Janko Leddin, Alexander Rommel, Elena von der Lippe, the BURDEN 2020 study group

**Affiliations:** 1 Department of Epidemiology and Health Monitoring, Robert Koch Institute, Berlin, Germany; 2 Department of Environmental Hygiene, German Environment Agency, Berlin, Germany

**Keywords:** Burden of disease, Redistribution, Cause of death, Ill-defined death, Mortality

## Abstract

**Background:**

The cause of death statistics in Germany include a relatively high share (26% in 2017) of ill-defined deaths (IDD). To make use of the cause of death statistics for Burden of Disease calculations we redistribute those IDD to valid causes of death.

**Methods:**

The process of proportional redistribution is described in detail. It makes use of the distribution of the valid ICD-codes in the cause of death data. We use examples of stroke, diabetes, and heart failure to illustrate how IDD are reallocated.

**Results:**

The largest increases in the number of deaths for both women and men were found for *lower respiratory infections*, *diabetes mellitus*, and *stroke*. The numbers of deaths for these causes more than doubled after redistribution.

**Conclusion:**

This is the first comprehensive redistribution of IDD using the German cause of death statistics. Performing a redistribution is necessary for burden of disease analyses, otherwise there would be an underreporting of certain causes of death or large numbers of deaths coded to residual or unspecific codes.

## Background

Globally, burden of disease (BoD) analyses are performed to assess the health state of populations [1]. Applying a standardized concept which covers all relevant health impairments allows comparing different diseases and injuries as well as related risk factors [2]. A key component of BoD studies is the summary measure of population health the *Disability-Adjusted Life Year* (DALY). DALY summarize the amount and severity of health problems experienced by a population and consider both fatal and non-fatal health outcomes [3]. The effect of the fatal health outcome is expressed by using the *Years of Life Lost*, the non-fatal health outcome is measured through *Years Lived with Disability* (YLD).

The calculation of the YLL is usually based on the cause of death (CoD) statistics, which use information from death certificates. In most cases the death certificate includes more than one cause and often the full chain of events leading to death. In Germany the CoD are classified according to the principles of the International Statistical Classification of Diseases and Related Health Problems (ICD-10, WHO Version). Besides the physician certifying the death, specialized coders in the statistical and health offices, as well as supporting software may influence the decision on the main, underlying CoD, that is then transferred to the national CoD statistics [4]. The underlying CoD should be the starting point of the chain of events leading to death. Secondary preceding causes and comorbidities recorded on the death certificate are not included in the nationally reported CoD statistics in Germany [5]. Furthermore, in some federal states the coding of the underlying CoD is done electronically through the implementation of specific software (Iris/MUSE). However, many federal states are still coding manually [6–9]. This may result in imprecisions.

Due to various reasons some ICD-10-codes in the CoD statistics are considered to be not sufficiently informative or valid for BoD estimations – sometimes referred to as garbage codes [10]. In the following, we refer to them as ill-defined (causes of) death or IDD. These codes describe conditions which cannot or should not be considered as an underlying CoD [11, 12]. Reasons for this might be missing information regarding the death cause or lack of training of the person coding the death [13]. A more detailed description on the major types of IDD can be found in Appendix 1.

Though, this phenomenon occurs in all CoD statistics worldwide the amount of the IDD varies largely across countries [14–16]. Comparing the shares of IDD in the CoD statistics for the years 2015 or 2016 from six countries with rather advanced health systems, Mikkelsen et al. revealed, that the share of IDD in Germany (26%) ranges between the shares found in Canada (22%) and in Japan (36%) [15]. Depending on the amount of IDD, the CoD statistics may not accurately reflect a country’s mortality, hampering comparisons or leading to biased priorities [17]. In consequence, as the underlying cause is not clearly identifiable large amounts of deaths with residual or unspecific codes may not be considered when deriving specific public health measures. Furthermore, the actual importance of certain CoD may be largely underestimated. IDD in this respect challenge BoD studies worldwide because a valid recording and reporting of CoD in a population is the basis for calculating YLL and hence BoD estimates.

The *Institute for Health Metrics and Evaluation* (IHME), responsible for the *Global Burden of Diseases, Injuries, and Risk Factors (GBD) Study*, has provided a thorough classification of the IDD which is updated with each cycle of the GBD study. Before calculating YLL these IDD are redistributed to valid CoD, using different statistical methods and employing profound and comprehensive algorithms [10, 16]. In general, the methods used for the redistribution in the GBD study include proportional reassignment, fixed proportions, regression models, experts’ opinions, and fractional assignment of a death assigned to multiple causes [10]. The World Health Organization developed a different classification of IDD [18], still resulting in a relatively high share of deaths (15.6% in 2015) classified as IDD in Germany [19].

Besides the GBD study, national BoD assessments are becoming increasingly available [20–22]. The project BURDEN 2020 – Burden of disease in Germany at national and regional level – is piloting a national BoD study for Germany [23]. The identification of IDD in this study follows the methodology provided by the GBD study ([16], personal communications with M. Naghavi 2019). Furthermore, part of the GBD study’s redistribution methods are adopted and applied to the German context. In contrast to the GBD study’s calculations for Germany, in BURDEN 2020 the YLL calculation and thus the IDD redistribution are performed not only on national but also on subnational level. The aim of the present article is to provide an in-depth description of the procedures for redistributing the IDD to valid codes in BURDEN 2020 and the impact this has on the case numbers for specific CoD in Germany.

## Methods

The German CoD statistics provide only one CoD, the underlying CoD. In BURDEN 2020 we therefore chose a redistribution method which refers to this one underlying CoD and makes use of the proportional distribution of deaths across valid ICD-codes. In general, 4 steps need to be taken to adjust the CoD statistics for calculating BoD: **1)** defining and grouping IDD in IDD packages (IDD redistributed together in the same processing step), **2)** defining valid target codes (reflecting the probable *true* underlying CoD) for each IDD package, **3)** deciding on and applying a redistribution methodology, **4)** structuring and grouping of ICD-codes to form suitable CoD. We follow the definition of IDD and the corresponding target codes (step 1 and 2) from the GBD study but chose our own approach for the redistribution (step 3). The grouping of ICD-10-codes to cause groups (step 4) has also been adapted from the GBD study. Hence, we shortly describe the four steps of utilizing CoD statistics for BoD estimations in the following, before we describe the complex redistribution processes (here step 3) in detail in the second part of the Methods section.

### IDD definition and grouping in IDD packages

The GBD study list of IDD contains more than 7000 ICD-10-codes (3- and 4-digit codes) of which 859 actually occur in the German CoD statistics in 2017. Overall, 932,269 deaths were registered in 2017 and 26% of all deaths were coded as IDD [19]. The IDD are grouped in IDD packages. This grouping is based on the medical association of certain ICD-codes and entails that all deaths belonging to one package are redistributed together following the same procedure. For each package a set of so-called target codes (see following section) is defined.

Depending on the type of IDD (impossible, intermediate, immediate, and unspecified cause; see Appendix 1), different objectives are pursued with the redistribution. Impossible IDD should be more generally allocated to various plausible CoD. The aim of handling sequela (intermediate and immediate causes) is to trace back to the underlying CoD. Unspecified causes should be transferred to more specific ones. In the correction process age, sex, and regional (place of residency) assignments are not changed.

### Definition of target codes for IDD

The IDD have to be reassigned to valid ICD-codes and consequently to valid CoD. The valid codes are called target codes and for each IDD package a set of target codes is defined. Target codes can be considered as the probable *true* underlying CoD in the case that a specific IDD was coded. BURDEN 2020 follows the GBD methodology in this step and thus, uses the same sets of target codes [16]. In general, the definition of the target codes requires understanding the pathology and epidemiology of the IDD. The specific target codes for each IDD package were made available to the BURDEN 2020 project through personal communication with Mohsen Naghavi as part of a memorandum of understanding between the Robert Koch Institute and IHME.

### Decision regarding redistribution method

**Fig. 1:**
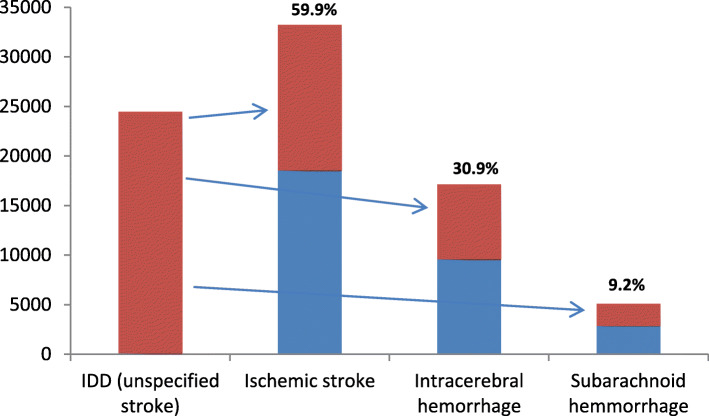
Example for the proportional redistribution of unspecified stroke

In BURDEN 2020 we apply a proportional redistribution method for relocating IDD. This means that the distribution of the IDD to target codes starts from the empirical proportion of valid codes as reported in the death register. In Fig. 1 the redistribution of the IDD is depicted by using an example. The target codes for the IDD *unspecified stroke* can be grouped in three specific stroke groups: *ischemic stroke*, *intracerebral hemorrhage*, and *subarachnoid hemorrhage*. In our example, ischemic stroke accounts for 59.9% of all valid codes. Accordingly, 59.9% of all IDD are redistributed to the group of ischemic strokes. Hence, the original proportion remains stable meaning that the distribution of valid codes is the same before (in blue) and after (in red) the redistribution. The main assumption in the proportional redistribution is, that the empirical distribution of target codes represents the actual distribution of CoD in the population. The redistribution with all necessary steps to be taken will be explained in detail in the following section (*Approach for the redistribution in BURDEN 2020*).

### Structuring and grouping of ICD-codes

**Fig. 2:**
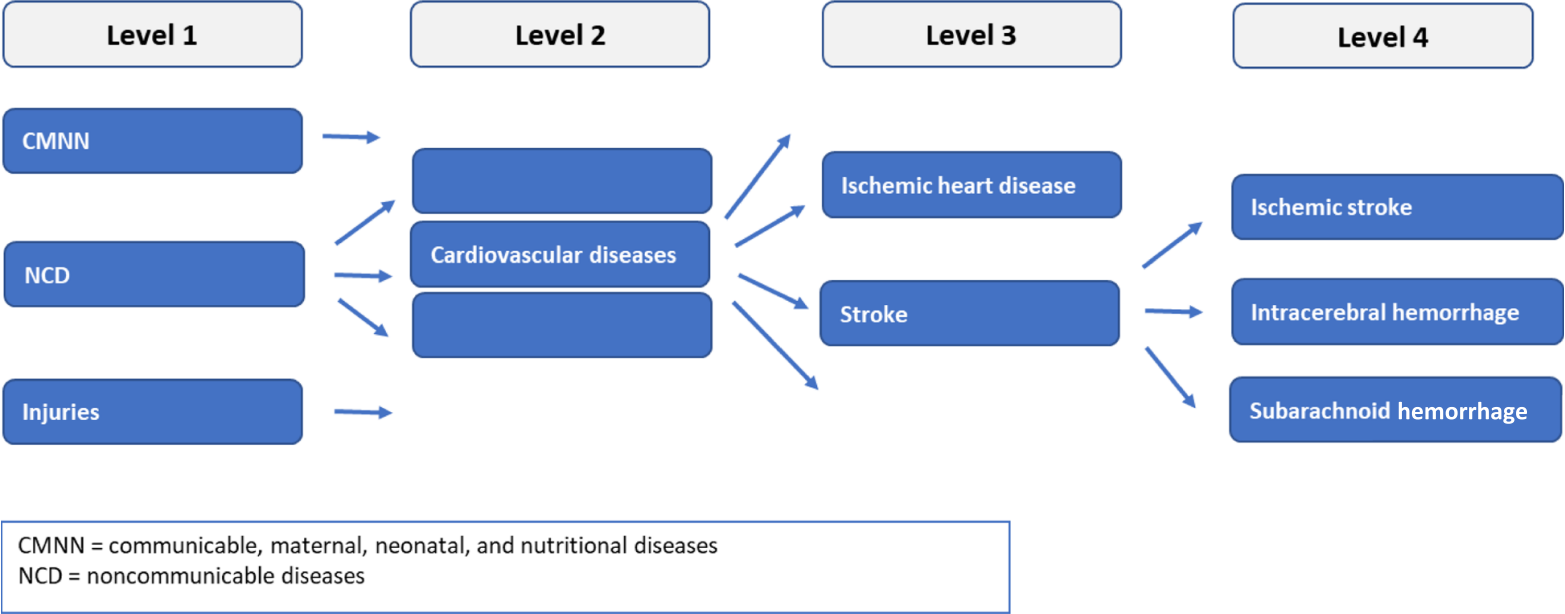
Causes of death hierarchy of the GBD study

The CoD statistics in Germany are available on a very detailed level of ICD-10 (four-digit codes). However, to get a comprehensive picture of CoD in Germany and to increase usability for public health concerns, this information needs to be aggregated and simplified. For this purpose, the hierarchical organization of the CoD at different levels, adopted from the GBD study, is implemented in BURDEN 2020 (Fig. 2) [16]. At the highest level (level 1), all valid ICD-10-codes are grouped into three broad cause categories: 1) Communicable, maternal, neonatal, and nutritional diseases (CMNN), 2) Non-communicable diseases (NCD), and 3) Injuries. Level 2 disaggregates these level 1 causes into 21 cause groups. NCD for example are subdivided into cardiovascular diseases, neoplasms, chronic respiratory diseases etc. (see Table A1 in Appendix 2). On level 3 cardiovascular diseases for example are further among others in ischemic heart disease or stroke. For some ICD-10-codes level 3 is the most detailed cause level. Where more detailed data are available or specific policy requirements exist a further disaggregation to level 4 is possible ([16], pages 1738–1739; personal communications with M. Naghavi 2019). Diabetes for example on level 4 is divided into diabetes mellitus type 1 and type 2. For stroke we can differentiate between ischemic stroke, intracerebral hemorrhage, and subarachnoid hemorrhage on level 4.

This cause hierarchy (see Fig. 2 and Table A1 in Appendix 2) also is the framework for presenting results on the disease burden related to cause specific mortality. It is comprehensive in the way that all valid ICD-codes are assigned to one exclusive cause on each level [16]. We adapted this GBD mapping of ICD-codes to CoD and refer to the different levels in the results section of this paper and other publications [24, 25].

#### Approach for the redistribution of IDD in BURDEN 2020

**Fig. 3:**
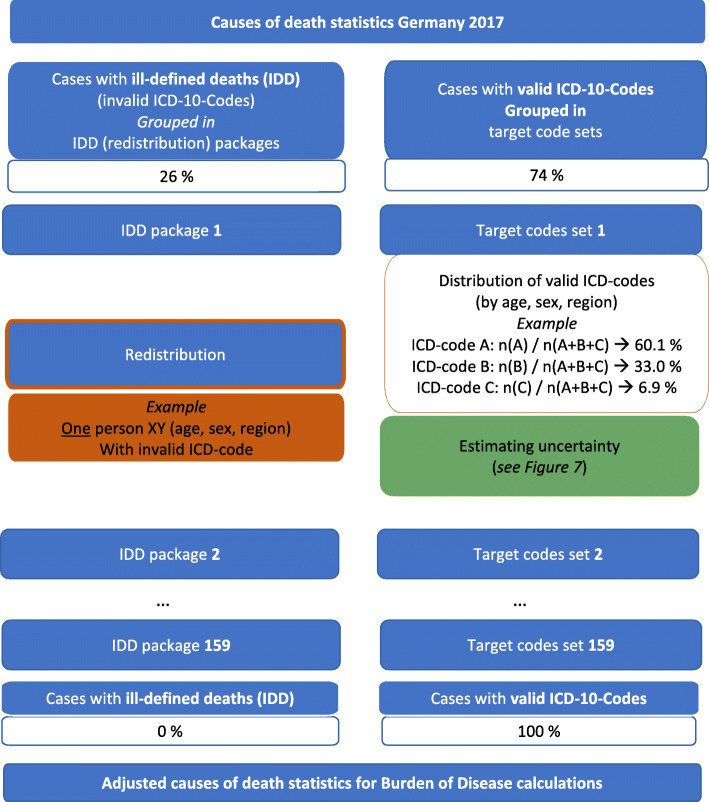
Consecutive process of redistributing IDD to valid ICD-codes

A step-by-step description of the redistribution procedure applied in BURDEN 2020 is presented in Fig. 3. The share of 26% IDD of all deaths in 2017 is grouped and assigned to target codes (see section before). As the IDD are grouped in 166 different packages (159 relevant for the German data), each package has a clearly assigned set of target codes whereas target codes are not exclusively assigned to only one package. Hence, specific codes can be assigned as target codes for several IDD packages.

The IDD packages are redistributed successively, shifting IDD to valid ICD-codes. In this way, the redistribution procedure is a stepwise reassignment. Consequently, the number of valid ICD-codes increases after the application of each package. Since more cases are defined as valid after each redistribution step, the number of cases forming the empirical distribution of valid target codes for the next step of the redistribution increases. Consequently, each redistributed package has an impact on the distribution of valid ICD-codes. Beyond the rational of using distinct packages, the redistribution is carried out age and sex specific. For instance, when reallocating unspecified stroke, which is considered an IDD, in women aged 82 we use the valid distribution of target codes within the group of women aged 80 to 84. Furthermore, the redistribution is performed on a subnational level (here federal states) which assures that regional variations in the CoD statistics are considered (see below).

#### Example for redistribution: stroke

**Fig. 4:**
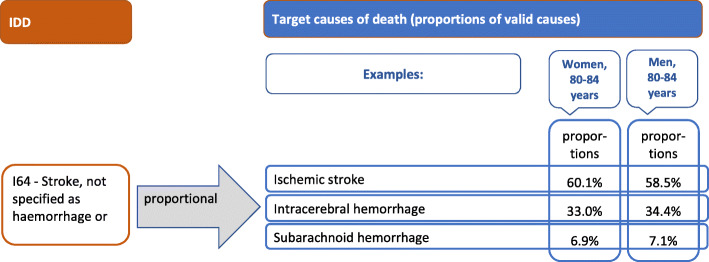
Example for the redistribution of unspecified stroke IDD in women and men aged 80 to 84

For most IDD packages a set of general target codes is defined which are neither age nor sex specific. One example outlined here is a package that encompasses all IDD belonging to the category of *unspecified stroke.* Different target ICD-codes are assigned to this package which can be grouped to three different causes: *ischemic stroke*, *intracerebral hemorrhage*, and *subarachnoid hemorrhage* (see Fig. 4). All IDD belonging to the unspecified stroke package are redistributed to the same target codes. However, the proportions of those target codes vary by age and sex. Figure 4 shows the causes containing the specific target codes for unspecified stroke and their proportions in women and men aged 80 to 84. Among women 60.1% of all cases with unspecified stroke are reassigned to ischemic stroke, 33.0% are moved to intracerebral hemorrhage, and 6.9% belong to subarachnoid hemorrhage after redistribution. For men in the same age group the proportions are 58.5, 34.4, and 7.1%, respectively.

#### Example for redistribution: gastrointestinal bleeding – with varying target codes

**Fig. 5:**
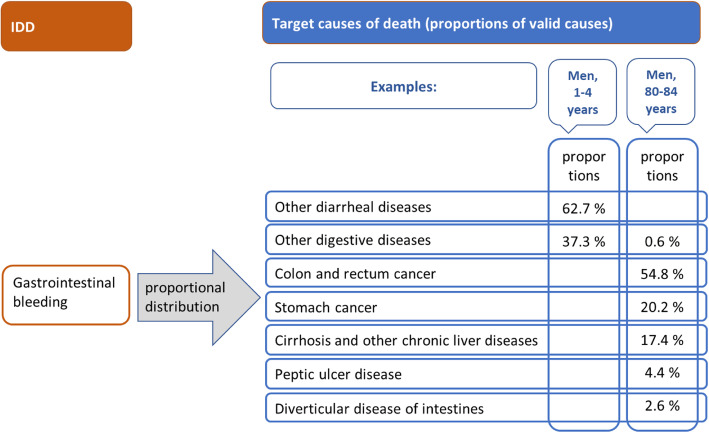
Example for the age specific redistribution of gastrointestinal bleeding

Some IDD packages have sets of target codes that vary by age or sex. *Gastrointestinal bleeding* is only reassigned to o*ther diarrheal diseases* and *other digestive disease* for deaths under 15 years of age (here illustrated for boys aged 1–4). However, for deaths at the age of 15 or older (here displayed for men aged 80–84) a larger set of target codes and resulting underlying CoD is assumed, including *colon and rectum cancer*, *stomach cancer*, and *cirrhosis and other chronic liver diseases* (see Fig. 5).

#### Subnational redistribution

**Fig. 6:**
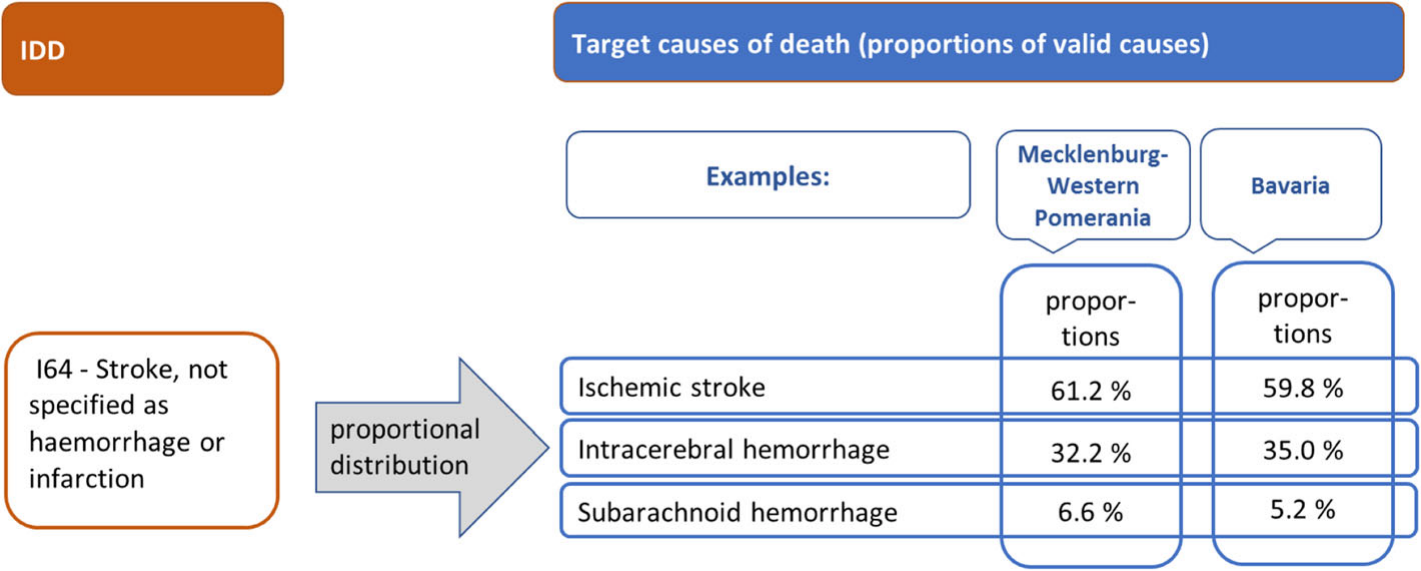
Example for the subnational redistribution of IDD in the federal states of Mecklenburg-Western Pomerania and Bavaria

Redistribution is not only carried out within each sex and age group but is also performed on a subnational level. In the project BURDEN 2020 [25] we aim to report BoD estimates for the 96 German spatial planning regions (SPR). However, in less populated SPR empirically only very few or no deaths may be assigned to the target codes, especially in younger age groups. This results in a distribution with missing target codes (e.g. 0% of cases), which makes a redistribution of IDD to those valid codes impossible. To overcome this problem, still taking regional variation into account, we chose the distribution of valid ICD-codes for the 16 federal states for subnational redistribution. Through the last place of residency of the deceased, we can identify the SPR as well as the federal state. Hence, for redistributing IDD age and sex specific empirical proportions of target codes on the federal state level are used (see example in Fig. 6). Additionally, however, the results can still be presented for each SPR.

#### Estimation of the uncertainty intervals

**Fig. 7:**
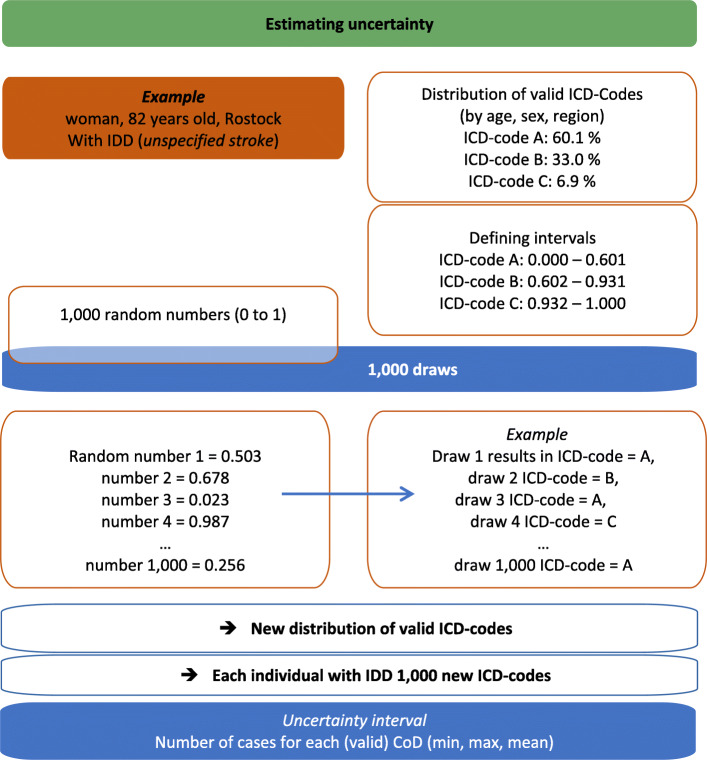
Example for estimating uncertainty when redistributing IDD to valid ICD-codes

Since each IDD has multiple target codes, we want to estimate the uncertainty that evolves from redistributing IDD to valid target codes. In this sense, the uncertainty interval (UI) defines the range of case numbers in which the true value is likely to lie. Thus, the uncertainty interval reflects that there are several potential target codes available. Additionally, it needs to be considered that the different redistribution packages vary largely with regard to the number and scope of target codes. Besides the number of IDD redistributed this influences the width of the UI. For each death coded as an IDD, the process of redistribution is repeated 1000 times (see Fig. 7) to allow for a variation of the possible actual CoD. In practice this is done by drawing 1000 random numbers between 0 and 1, which can be interpreted as proportions (see example below). Thereafter, we use the specific distribution of target codes for reassigning ICD-codes (Fig. 7).

Stroke is used as an example to present the process quantifying uncertainty. An unspecified stroke can be assigned to ischemic stroke, intracerebral, or subarachnoid hemorrhage. These three causes of death are distributed differently in the population, thus, occurring with different frequencies in the CoD statistics. These distributions additionally vary by age, sex, and region.

Referring to stroke, the distribution for women (aged 80 to 84), which was 60.1% *ischemic stroke*, 33.0% *intracerebral hemorrhage*, and 6.9% *subarachnoid hemorrhage* is redefined in intervals. Using these proportions, the random numbers can be used for reassigning new ICD-codes. Random numbers between 0 and 0.601 are assigned to the cause ischemic stroke, values between 0.601 and 0.931 to intracerebral hemorrhage, and values between 0.931 and 1.000 to subarachnoid hemorrhage. For each of the 1000 random numbers a new valid ICD-code is assigned to the identified IDD. A higher proportion of a target code results in a higher probability for assignment to this code.

Naturally, the 74% valid ICD-Codes (see Fig. 3) are not changed and remain as they are. They are however replicated 1000 times and succeedingly joined with the redistributed deaths. After redistribution, this results in 1000 valid ICD-codes for each of the 932,269 deaths in 2017. The uncertainty intervals can be derived by using the minimum, maximum, and mean number of deaths for a specific ICD-code with respect to the resulting distribution across the 1000 draws. Thereafter, the ICD-codes are grouped to CoD on different levels (see Fig. 2) and this results in uncertainty intervals for all valid CoD. Additionally, the 1000 draws can be used to depict uncertainty when calculating YLL [24].

## Results

In the first part of the results section, we want to illustrate how the redistributed cases are reallocated from IDD to valid causes, looking at specific examples. In the second part, we take a broader perspective and compare all cases before and after redistribution by cause level.

### Examples: heart failure, stroke, and diabetes

#### Heart failure redistribution

One of the biggest groups of IDD in Germany is heart failure. Physicians often choose heart failure as underlying CoD which results in 39,300 cases in the German CoD statistics in 2017 [14]. Heart failure is defined as an IDD because it cannot be the underlying CoD but is rather the consequence of an underlying cause (e.g. ischemic heart disease). Nevertheless, this code is largely used as a main CoD, hampering the use of CoD statistics for BoD analyses.

**Fig. 8:**
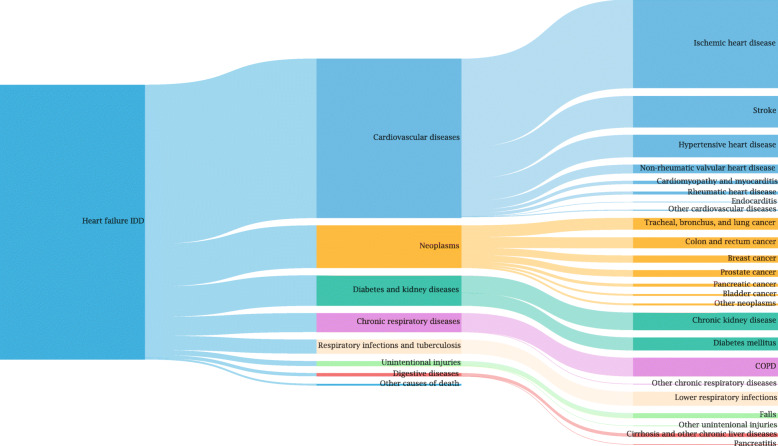
Redistribution of cases classified as heart failure IDD to valid causes (level 2 and 3)

The defined target codes for heart failure are spread across the whole ICD catalogue (Fig. 8). Of the 39,300 heart failure cases on average almost 22,800 cases are redistributed to *cardiovascular diseases*, 6000 to *neoplasms*, and 4300 to *diabetes and kidney diseases*. 2700 cases are redistributed to *chronic respiratory diseases* and 2000 to *respiratory infections and tuberculosis*. For 2017, the largest cause group on level 2 is cardiovascular diseases of which on level 3 the largest causes are *ischemic heart disease*, *stroke*, and *hypertensive heart disease*. All heart failure IDD reallocated to neoplasms on level 2, are mainly composed of *tracheal, bronchus, and lung cancer*, *colon and rectum cancer*, as well as *breast cancer* on level 3.

#### Stroke redistribution

**Fig. 9:**
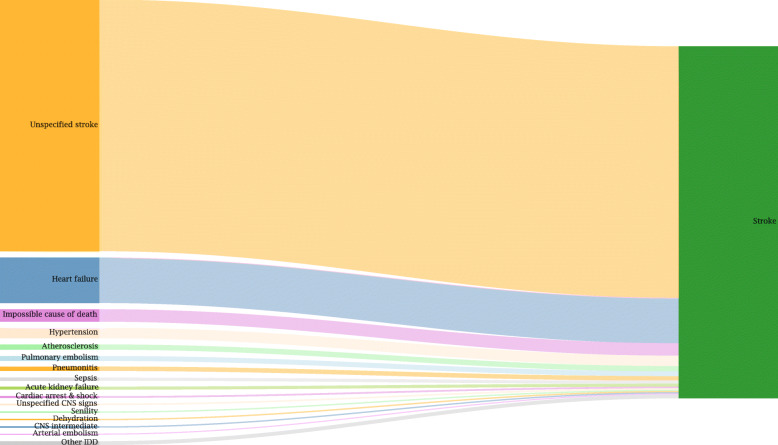
Redistribution of cases classified as IDD to stroke (level 3)

Besides observing which are the biggest groups of target codes for a specific IDD, it is also interesting to analyze which IDD contribute most to a particular valid CoD. Below is the example for stroke as a cause showing a significant increase by 34,200 deaths after redistribution (Fig. 9).

The data for 2017 show that the IDD group of *unspecified stroke,* that includes 24,500 cases, on average contributes most to the increase of stroke as a valid CoD. Thereafter, *heart failure* contributes around 4400 cases, the *impossible CoD* 1200, and *hypertension* 1000. As a result, the number of deaths from stroke has increased from 30,975 cases to 65,218 (see Table 3).

#### Diabetes redistribution

**Fig. 10:**
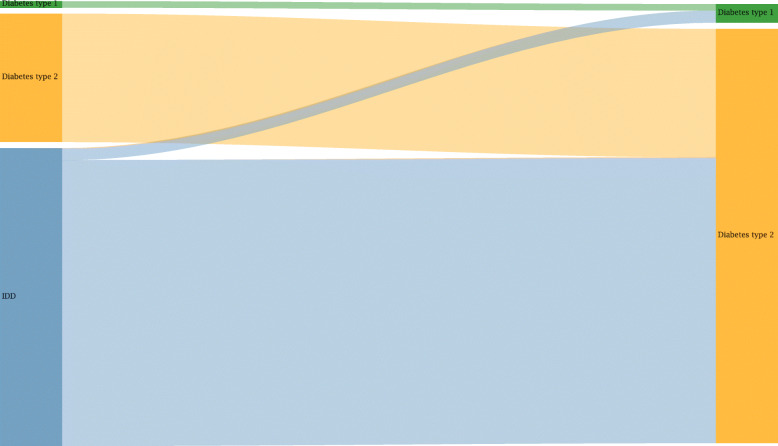
Diabetes type 1 and 2 (level 4) before and after redistribution of cases classified as IDD

Diabetes is another CoD where the number of deaths increases largely due to redistribution, especially since for a large amount of cases unspecified diabetes is recorded on the death certificates. Figure 10 indicates that the increase of diabetes cases after redistribution is around threefold. Whereas, the heart failure (Fig. 8) and stroke (Fig. 9) examples put emphasis on the redistributed cases, Fig. 10 explicitly includes valid cases before and after redistribution. Fig. A1 in the Appendix 3 additionally depicts the precise IDD groups contributing to diabetes type 1 and 2. Besides the already stated largest group of *unspecified diabetes*, IDD belonging to the groups of *chronic kidney disease due to unspecified type*, *heart failure*, and *impossible CoD* contribute most to diabetes.

### Cases before and after redistribution

#### Cases before and after redistribution on level 1 and 2

On level 1 all CoD are divided into three groups. Before redistribution 10,091 deaths are assigned to *communicable, maternal, neonatal, and nutritional diseases* (CMNN: 1.5%), 649,658 to *non-communicable diseases* (NCD: 93.9%) and 31,718 to *injuries* (4.6%). After redistribution 36,930 cases are defined as CMNN (4.0%), 850,534 (91.2%) as NCD and 44,805 (4.8%) as injuries (Table 1). The number of cases with CMNN as CoD displays a threefold increase. This results in 4.0% of all deaths being assigned to this group instead of 1.5% before redistribution. The share of NCD on the other hand decreases in the process of redistribution from 93.9 to 91.2%. However, by numbers of deaths it remains by far the largest group.

**Table 1:** Cases by causes of death – Level 1, before and after redistribution of cases with ill-defined causes of death

	Before redistribution	Share	After redistribution	Share
Communicable, maternal, neonatal, and nutritional diseases	10,091	*1.5%*	36,930	*4.0%*
Non-communicable diseases	649,658	*93.9%*	850,534	*91.2%*
Injuries	31,718	*4.6%*	44,805	*4.8%*
Ill-defined ICD-codes	240,802			
**Total**	**932,269**		**932,269**	

Source: causes of death statistics, Germany, 2017, own calculations

**Fig. 11:**
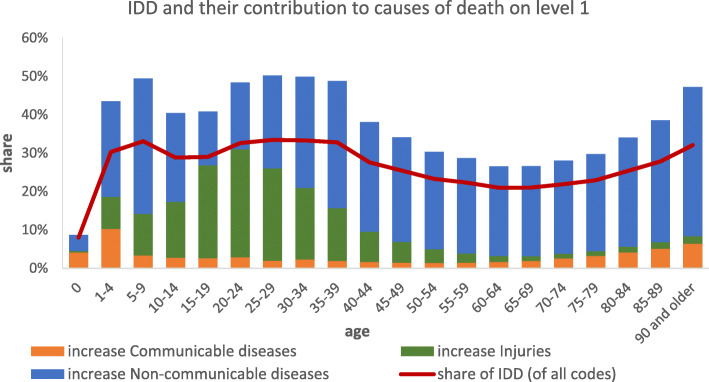
Redistribution of cases classified as IDD to Diabetes mellitus tType 1 and 2 on level 4, all IDD packages contributing

In Fig. 11 we additionally examined the share of IDD across age groups and to what degree the three main groups of causes of death (level 1) increase after redistribution. Since most deaths belong to NCD they also increase most in absolute numbers in the process of redistribution but variations across age can also be observed. For deaths registered in the younger age-groups, aged 1–4, we especially see a high increase of CMNN. Between 15 and 25 years of age injuries show the highest increase after redistribution. For all other age groups NCD contribute most to the increase of case numbers.

On level 2 the ranking of the top three CoD – *cardiovascular diseases, neoplasm*, and *neurological diseases* – remains unchanged (Table 2). In contrast, after redistribution *diabetes and kidney diseases* move two ranks up to the forth position. *Chronic respiratory diseases* and *digestive diseases* both decrease in one rank after redistribution. Apart from that, the effect of the redistribution can be evaluated looking at the percent of increase. *Cardiovascular diseases* and *neoplasms* cause the most deaths in Germany and account for 64.5% of all deaths. Irrespective of the unchanged ranking for these conditions with 42% and 25% respectively, we nevertheless observe a large increase in case numbers after redistribution. The highest increases, however, can be shown for CoD which are probably underreported: *respiratory infections* (1135% increase) and *diabetes and kidney diseases* (79% increase). The increase in respiratory infections mostly results from unspecified pneumonias being reallocated. Also, the number of *HIV/AIDS and sexually transmitted infections* increases significantly (235% increase), though this in general is only causing a small number of deaths.

**Table 2:**
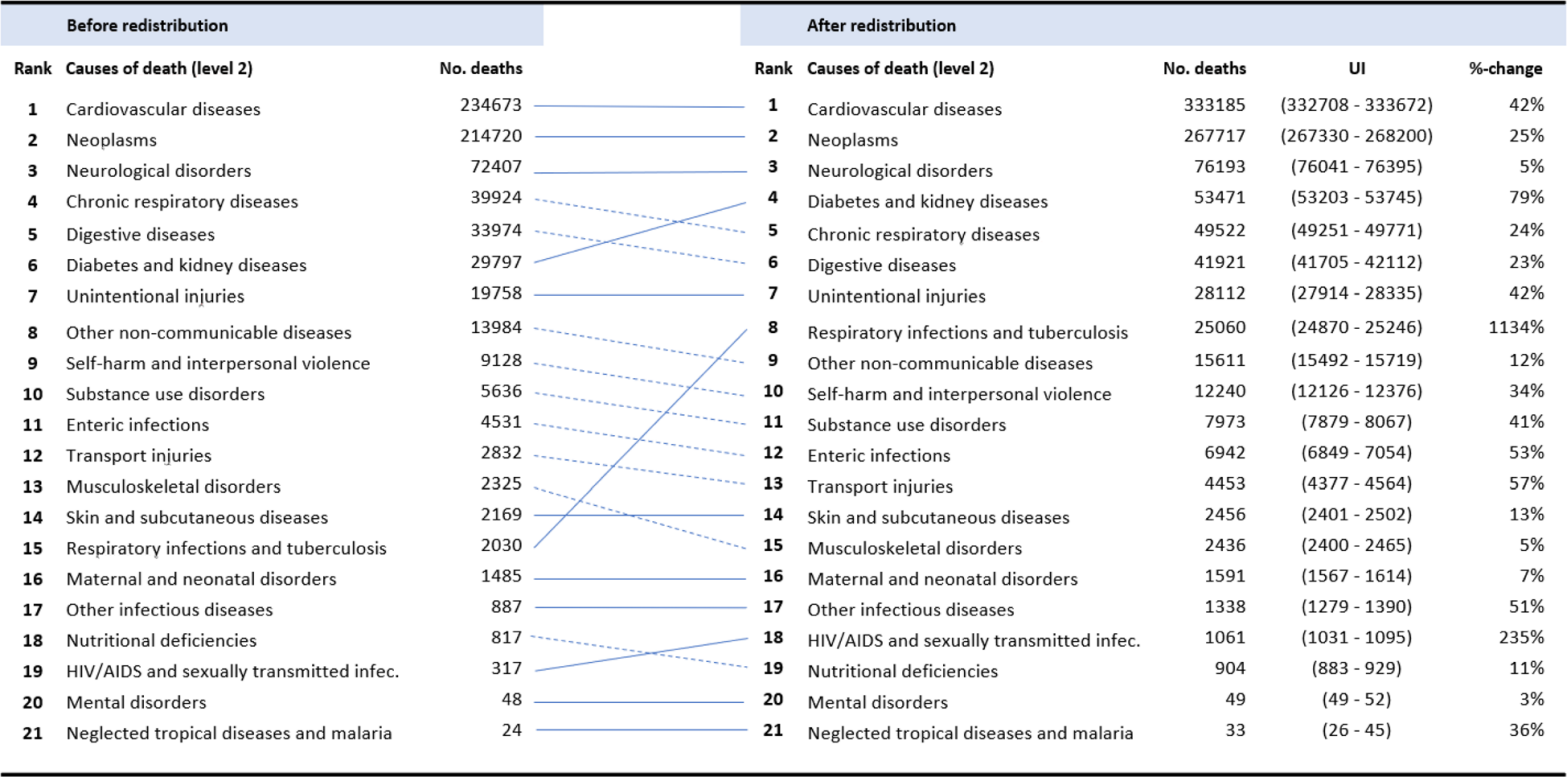
Deaths by causes on level 2 before and after redistribution of IDD

#### Top 20 causes of death (level 3) before and after redistribution

In Table 3 the 20 most frequent CoD on level 3 before and after the redistribution of the IDD are presented. For women the top three CoD after redistribution are *ischemic heart disease*, *stroke*, and *Alzheimer’s disease and other dementias*. For men these are *ischemic heart disease*, *tracheal, bronchus and lung cancer*, and *stroke*.

**Table 3:**
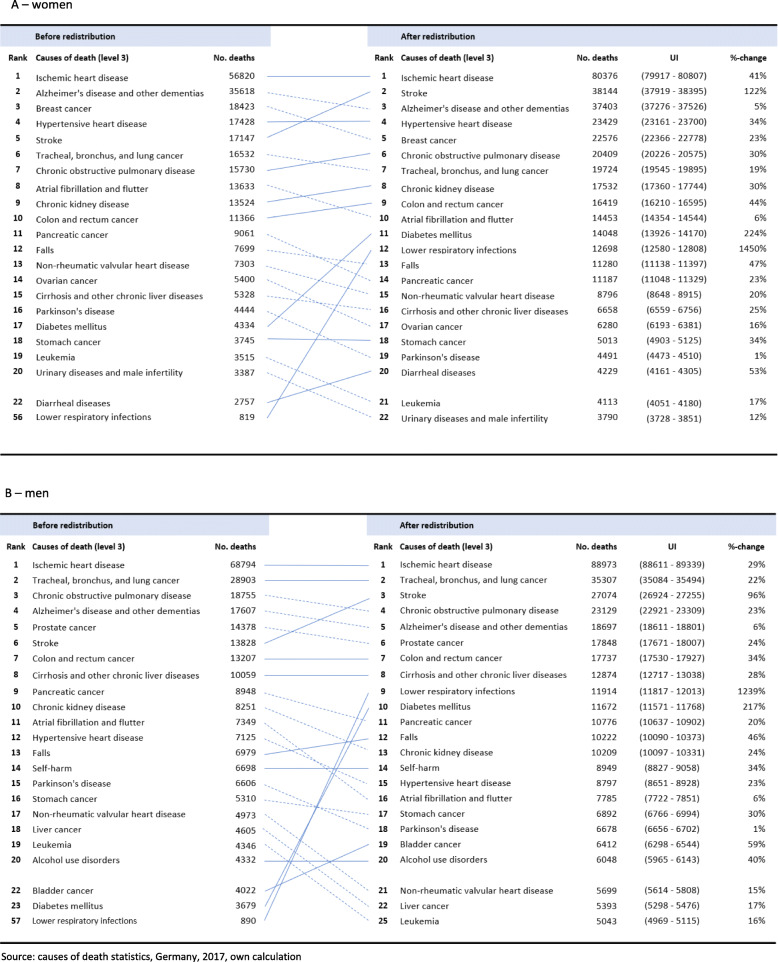
The 20 most frequent causes of death, for women (A) and men (B) before and after redistribution (level 3)

As before, we observe quite large variation in the percent of increase in case numbers after redistribution. The largest increases for both women and men can be found for *lower respiratory infections* (+ 1450% and + 1239%, respectively), *diabetes mellitus* (+ 224% and + 217%), and *stroke* (+ 122% and + 96%). As indicated before lower respiratory infections increase to such extent due to the great amount of unspecified pneumonias in the data.

#### Regional variations

The number of IDD varies largely across the German federal states and spatial planning regions (SPR), ranging from 14% to 36% IDD in 2015 [19]. This influences the width of the uncertainty intervals (UI). For example, for ischemic heart disease deaths in men between 80 and 94 years of age, the case numbers more than double after redistribution in the region with the highest share of IDD (262, UI: 214–308). In the region with the lowest share of IDD the case numbers only increase by 10% (672, UI: 639–705) which results in a much smaller UI (5% deviation from mean to minimum and maximum) than for the first region (18% deviation from mean in both directions). More results on the regional variation of number of deaths and YLL will soon become available through another publication [24] and a website [25].

## Discussion

In this paper the method of redistributing IDD to valid CoD, as applied in the German BoD study BURDEN 2020, has been illustrated. Whereas some parts of the method like the identification of IDD and target codes were adopted from the GBD study, the redistribution and the approach to calculate uncertainties were necessary adjustments. Our aim was to depict how CoD statistics can be made suitable for BoD estimations and beyond. Since 26% of all deaths in Germany in 2017 are defined as IDD, we observe considerable changes in case numbers before and after redistribution. For the specific CoD stroke, diabetes, and respiratory infections the numbers more than double.

The method used for the redistribution is in part an adaptation of the GBD method, adjusted to the German data. The results show that there are some significant differences, when comparing the final CoD data. The GBD study (2017) estimated about 15,000 more deaths in 2017 in Germany than reported in the German national CoD statistics [16]. This difference is most likely due to the additional correction steps that IHME applies when assessing the data quality and population coverage. The difference in the total number of deaths is reflected also in the cause specific number of deaths. However, the proportions of the main groups of CoD are very much comparable between the GBD and the German BURDEN 2020 study.

The development and implementation of methodologies for redistributing IDD is also done in other countries [21, 22, 26, 27]. The methods applied mainly reflect the available country specific data. Accordingly, the handling of IDD in national BoD studies differs significantly. In Scotland the CoD statistics include information on multiple CoD and in some cases, deaths can be linked to individual clinical records. Thus, it is possible to develop a more precise, country specific method of identifying and redistributing IDD [28]. Likewise, Australia has developed its own algorithms for redistributing IDD. It includes several methods such as data linkage for obtaining additional information, usage of multiple CoD statistics, and proportional redistribution [29]. Other countries that lack multiple CoD statistics are forced to rely on alternative methods. For instance, Brazil has performed further research based on information from different health service providers or verbal autopsy. In cases where the actual CoD was not possible to be defined a proportional redistribution method was applied [26]. In the Netherlands experts pursue a one-number-policy, they do not redistribute IDD but instead use the CoD statistics without adjustment [30].

The above described method of redistribution is applied on the federal state level. It must be considered that the subnational differences in mortality registration procedures influence the quality of the data and the regional amount of IDD [19, 31]. In regions with higher shares of IDD we see more variation in the CoD. Hence, we observe broader uncertainty bands, since they are estimated only for the redistributed cases, which have varying target codes. Valid ICD-codes remain the same through the process. Thus, the uncertainty bands are of importance additionally depicting the variation in quality of CoD registration between the regions.

### Limitations and strengths

The adopted method used the definition of IDD as developed by IHME as part of the GBD study. However, it must be considered that in many cases scientists and physicians may have different perspectives on which diseases should be defined as IDD. For instance, for some CoD there is no consensus whether the condition should be classified as underlying CoD or as a sequela of another disease, the second being an intermediate or secondary CoD. A critical example is septicemia, which is considered an IDD in the GBD study, with its own redistribution package. This assessment is controversially discussed, as some experts see septicemia, at least for a part of the reported deaths, as the underlying CoD [32].

Another limitation of the study is the lack of multiple CoD data. The redistribution methodology in Germany could be largely improved should such data become available. Further research is underway to test possible redistribution methods using multiple CoD data for some regions in Germany. Related to this, a further limitation of the applied method is the assumption that the valid CoD present the *true* distribution of valid codes. Since currently no other CoD data are available, there is no possibility to verify the distribution. Considering the low number of autopsies in Germany [33] and the fragmented use of electronic coding of deaths [9] some inaccuracies are possible.

Advantageously, the applied redistribution method is transparent and comprehensible. Another strength of the study is the high quality of the German mortality data, especially with regard to registering the correct number and the age and sex of the deceased. In Germany almost full coverage of all deaths can be assumed and hence no methods for correction of possible underreporting needs to be applied. For many other countries, where mortality data do not have the same quality, and consequently a lower coverage, the GBD study has developed methods for corrections [16].

Only by redistributing IDD to valid ICD-Codes the CoD data can be fully used for burden of disease calculations. To depict the varying target codes uncertainty intervals supporting the interpretation of results are provided indicating the margin in which the actual death counts may vary. Another strength of the study, is the redistribution of IDD on a subnational level. As shown before [19] the quality of the CoD statistics in Germany differs strongly between the federal states. Additionally, we generally expect and observe differing mortality patterns across the federal states [34, 35], e.g. due to differences in age structure and socio-economic status [36, 37].

### Future directions

The method described here reflects the availability of CoD data in Germany. It is the first comprehensive redistribution of IDD within the CoD statistics for Germany. Further methodological developments are possible. We have only analyzed data from 1 year (2017). Combining years of data (3-year or 5-year period) might reduce random variations in the CoD data. Other areas of potential improvement include the usage of multiple CoD data which will allow a better determination of the target codes and the redistribution proportions. Furthermore, the selection of the target codes needs better documentation and possibly revision in the future. At the moment the selection of the target codes is based on current research and expert assessment provided by IHME, which is not always clearly documented or described.

Performing a redistribution method on the CoD statistics is currently very important for burden of disease analyses. Otherwise there would be an underreporting of certain CoD or large numbers of deaths coded to residual or unspecific codes. However, more efforts should be put into obtaining a better quality of death registries and hence CoD data in Germany. This encompasses defining the underlying CoD as well as providing information on the accompanying diseases [31]. The nationwide implementation of the Iris/MUSE software, which improves the electronic processing and correction of CoD data, is a step in that direction and will contribute to better recording of the underlying CoD [8, 9]. From a public health perspective these successive improvements are of large importance as CoD data are an important information base for the identification of health needs, the prioritization of actions required, and the development of targeted interventions.

## Figures and Tables

**Fig. 12:**
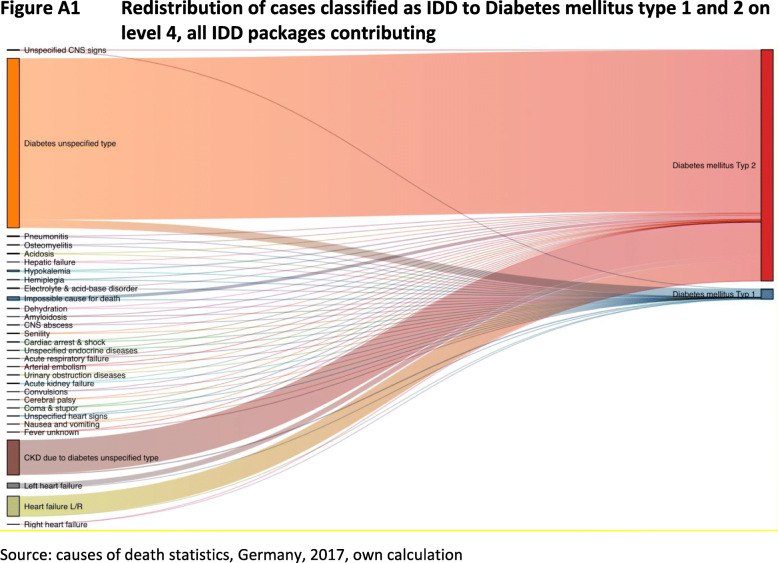
The increase of case numbers after redistribution by causes of death – Level 1 and the share of IDD, across age groups

## Data Availability

The datasets generated during the current study are not publicly available due to confidential information from IHME (Institute of Health Metrics and Evaluation). Data are however available from the authors upon reasonable request and with permission of IHME. The basis of our analysis are the German causes of death data. Without regional assignments and aggregated they are available as an excel file through the German Federal Statistical Office (https://www.destatis.de/DE/Themen/Gesellschaft-Umwelt/Gesundheit/Todesursachen/_inhalt.html Excel: *Ergebnisse der Todesursachenstatistik für Deutschland - Ausführliche vierstellige ICD10-Klassifikation – 2017.xlsx*).

## Appendix 1

**Types of IDD**

Four major types of IDD can be described: a) the causes that cannot or should not be used as underlying causes (impossible causes), b) the intermediate, c) the immediate and d) the unspecified causes [10].

The major part of IDD can be classified as (a) impossible CoD. This group comprises codes associated with morbidity but without the possibility of fatal outcomes. Examples are W50 Vitamin A deficiency or H10 - Conjunctivitis. Another grouping of impossible causes are R-codes which cover signs and symptoms but not directly a specific disease or injury and thus are not qualified to capture an underlying CoD. Examples for this group are R22.1 Localized swelling, mass and lump, neck or R53 Malaise and fatigue. Furthermore, implausible combinations of conditions with sex- or age information must be corrected.

Beside the impossible causes also (b) intermediate or (c) immediate causes which follow the actual underlying cause on the pathway of events leading to death are classified as IDD. Intermediate causes, such as heart failure, are clearly defined clinical entities that have always a precipitating event, which is the underlying CoD [10]. Immediate CoD are the final steps in the chain of events leading to death. Disseminated intravascular coagulation [defibrination syndrome] (D65) is one example of this type of IDD [10]).

Finally, (d) causes not clearly coded or classified are considered IDD within the GBD. The names of those codes typically include terms such as unspecified, other or with undetermined intent. The last one only applies to the category of injuries. Examples of this category are Y21 – Drowning and submersion, undetermined intent or C74.9 Endocrine gland, unspecified among the category malignant neoplasms.

## Appendix 2

**Table A1:** Mapping level 1 to 3 (Source IHME/GBD 2019, own depiction)

Level 1	Level 2	Level 3	
**Communicable, maternal, neonatal, and nutritional diseases**	Enteric infections	Diarrheal diseases	
		Invasive Non-typhoidal Salmonella (iNTS)	
		Other intestinal infectious diseases	
		Typhoid and paratyphoid	
	HIV/AIDS and sexually transmitted infections	HIV/AIDS	
		Sexually transmitted infections excluding HIV	
	Maternal and neonatal disorders	Neonatal disorders	
		Maternal disorders	
	Neglected tropical diseases and malaria	Other neglected tropical diseases	
		Cystic echinococcosis	
		Malaria	
		Chagas disease	
		Schistosomiasis	
	Nutritional deficiencies	Protein-energy malnutrition	
		Other nutritional deficiencies	
		Dietary iron deficiency	
	Other infectious diseases	Other unspecified infectious diseases	
		Encephalitis	
		Meningitis	
		Varicella and herpes zoster	
		Acute hepatitis	
		Whooping cough	
		Measles	
		Rubella	
	Respiratory infections and tuberculosis	Lower respiratory infections	
		Tuberculosis	
		Upper respiratory infections	
		Otitis media	
**Non-communicable diseases**	Cardiovascular diseases	Ischemic heart disease	Rheumatic heart disease
		Stroke	Aortic aneurysm
		Hypertensive heart disease	Peripheral artery disease
		Atrial fibrillation and flutter	Other cardiovascular and circulatory diseases
		Non-rheumatic valvular heart disease	Endocarditis
		Cardiomyopathy and myocarditis	Primary pulmonary arterial hypertension
	Chronic respiratory diseases	Chronic obstructive pulmonary disease	Pneumoconiosis
		Interstitial lung disease and pulmonary sarcoidosis	Other chronic respiratory diseases
		Asthma	
	Diabetes and kidney diseases	Chronic kidney disease	Acute glomerulonephritis
		Diabetes mellitus	
	Digestive diseases	Cirrhosis and other chronic liver diseases	Other digestive diseases
		Paralytic ileus and intestinal obstruction	Pancreatitis
		Vascular intestinal disorders	Inflammatory bowel disease
		Upper digestive system diseases	Inguinal, femoral, and abdominal hernia
		Gallbladder and biliary diseases	Appendicitis
	Mental disorders	Eating disorders	
	Musculoskeletal disorders	Other musculoskeletal disorders	Rheumatoid arthritis
	Neoplasms	Tracheal, bronchus, and lung cancer	Other malignant neoplasms
		Colon and rectum cancer	Malignant skin melanoma
		Breast cancer	Other pharynx cancer
		Pancreatic cancer	Lip and oral cavity cancer
		Prostate cancer	Uterine cancer
		Stomach cancer	Cervical cancer
		Bladder cancer	Soft tissue and other extraosseous sarcomas
		Leukemia	Larynx cancer
		Kidney cancer	Mesothelioma
		Liver cancer	Non-melanoma skin cancer
		Non-Hodgkin lymphoma	Thyroid cancer
		Brain and central nervous system cancer	Malignant bone tumors
		Esophageal cancer	Hodgkin lymphoma
		Ovarian cancer	Eye cancer
		Multiple myeloma	Testicular cancer
		Other neoplasms	Nasopharynx cancer
		Gallbladder and biliary tract cancer	Neuroblastoma and other peripheral nervous cell tumors
	Neurological disorders	Alzheimer’s disease and other dementias	Motor neuron disease
		Parkinson’s disease	Other neurological disorders
		Epilepsy	Multiple sclerosis
	Other non-communicable diseases	Urinary diseases and male infertility	Hemoglobinopathies and hemolytic anemias
		Endocrine, metabolic, blood, and immune disorders	Sudden infant death syndrome
		Congenital birth defects	Gynecological diseases
	Sense organ diseases	Other sense organ diseases	
	Skin and subcutaneous diseases	Bacterial skin diseases	Other skin and subcutaneous diseases
		Decubitus ulcer	Scabies
	Substance use disorders	Alcohol use disorders	Drug use disorders
**Injuries**	Self-harm and interpersonal violence	Self-harm	
		Interpersonal violence	
		Executions and police conflict	
		Conflict and terrorism	
	Transport injuries	Road injuries	
		Other transport injuries	
	Unintentional injuries	Falls	
		Adverse effects of medical treatment	
		Foreign body	
		Drowning	
		Fire, heat, and hot substances	
		Exposure to mechanical forces	
		Environmental heat and cold exposure	
		Other unintentional injuries	
		Poisonings	
		Animal contact	
		Exposure to forces of nature	
